# Biomedical Applications of Plant Extract-Synthesized Silver Nanoparticles

**DOI:** 10.3390/biomedicines10112792

**Published:** 2022-11-02

**Authors:** Sohail Simon, Nicole Remaliah Samantha Sibuyi, Adewale Oluwaseun Fadaka, Samantha Meyer, Jamie Josephs, Martin Opiyo Onani, Mervin Meyer, Abram Madimabe Madiehe

**Affiliations:** 1 Department of Science and Innovation (DSI)/Mintek Nanotechnology Innovation Centre (NIC), Biolabels Research Node, Department of Biotechnology, University of the Western Cape, Bellville 7535, South Africa; 2 Nanobiotechnology Research Group, Department of Biotechnology, University of the Western Cape, Bellville 7535, South Africa; 3 Health Platform Diagnostic Unit, Advanced Materials Division, Mintek, Randburg 2194, South Africa; 4 Department of Biomedical Sciences, Faculty of Health and Wellness Sciences, Cape Peninsula University of Technology, Bellville 7535, South Africa; 5 Organometallics and Nanomaterials, Department of Chemical Sciences, University of the Western Cape, Bellville 7535, South Africa

**Keywords:** anti-microbial activity, anti-cancer activity, anti-angiogenesis activity, green synthesis, nanotechnology, metallic nanoparticles, phytochemicals, phytonanotechnology, plant-synthesized AgNPs, silver nanoparticles

## Abstract

Silver nanoparticles (AgNPs) have attracted a lot of interest directed towards biomedical applications due in part to their outstanding anti-microbial activities. However, there have been many health-impacting concerns about their traditional synthesis methods, i.e., the chemical and physical methods. Chemical methods are commonly used and contribute to the overall toxicity of the AgNPs, while the main disadvantages of physical synthesis include high production costs and high energy consumption. The biological methods provide an economical and biocompatible option as they use microorganisms and natural products in the synthesis of AgNPs with exceptional biological properties. Plant extract-based synthesis has received a lot of attention and has been shown to resolve the limitations associated with chemical and physical methods. AgNPs synthesized using plant extracts provide a safe, cost-effective, and environment-friendly approach that produces biocompatible AgNPs with enhanced properties for use in a wide range of applications. The review focused on the use of plant-synthesized AgNPs in various biomedical applications as anti-microbial, anti-cancer, anti-inflammatory, and drug-delivery agents. The versatility and potential use of green AgNPs in the bio-medicinal sector provides an innovative alternative that can overcome the limitations of traditional systems. Thus proving green nanotechnology to be the future for medicine with continuous progress towards a healthier and safer environment by forming nanomaterials that are low- or non-toxic using a sustainable approach.

## 1. Introduction

Nanotechnology is a thriving field of science that incorporates materials at a nanoscale ranging in size from 1–100 nm [1,2]. Due to their small size, these nanoparticles (NPs) have unique physiochemical properties and exhibit extraordinary activities [3] with the potential to solve most of the health challenges faced by the globe. Metallic nanoparticles (MNPs) are among the nanomaterials that have been broadly used in the biomedical fields [4] to fight against infectious and chronic diseases. MNPs have admirable physiochemical properties such as magnetic, catalytic, photochemical, and mechanical attributes [5] that ensure and improve cellular response towards treatments [6].

MNPs are synthesized by using various metals such as gold, silver, iron, zinc, copper, palladium, platinum, and metal oxides [4,7]. However, more attention has been focused on the health-related activities of AgNPs [8]. The bio-activities of AgNPs are associated with their physical, chemical, and biological characteristics stemming from their shape, size, composition, and crystallinity compared with the bulk material [4]. Initially, AgNPs attracted global attention due to their anti-microbial activities [8] and are now widely found in commercial products, such as food packaging, soaps, cosmetics, plastics, and textiles [9,10]. However, the chemically synthesized AgNPs (cAgNPs) used in these products, together with their by-products, can be toxic and harmful to humans [11] and the environment [12,13]. In an effort to counteract these limitations, green synthesis methods were used to produce biogenic AgNPs that are biocompatible and have reduced bystander toxic effects [14]. Herein, the different approaches used for the synthesis of AgNPs were discussed with a strong focus on the plant-extract-synthesized AgNPs. Plant-synthesized AgNPs present a fresh perspective and a sustainable approach for the development of improved or new therapeutic strategies. AgNPs can be synthesized from easily accessible and renewable plant materials such as vegetables [15], fruits [16], and medicinal plants [17,18]. The phytochemicals in the extracts serve as reducing and capping agents [19] and are responsible for the stabilization of the AgNPs and their bioactivities. The phytochemicals as the capping agents will promote the biocompatibility of the AgNPs by preventing the Ag^+^ ions from leaching out. Moreover, the plant-synthesized AgNPs demonstrated similar and improved bioactivities to those of the cAgNPs, such as anti-microbial [20], anti-angiogenesis [21,22], anti-cancer [23] and anti-diabetic [24,25] agents. As such, they can be used to replace the cAgNPs found in consumer products and ones in clinical trials. The biocompatibility demonstrated by the green AgNPs suggests the possibility of their application in health as therapeutic agents. Exposure to AgNPs is inevitable as they are already used for both cosmetic- and health-related purposes, and the use of biogenic AgNPs can offer some form of confidence in using these products.

## 2. Synthesis of AgNPs

**Figure 1:**
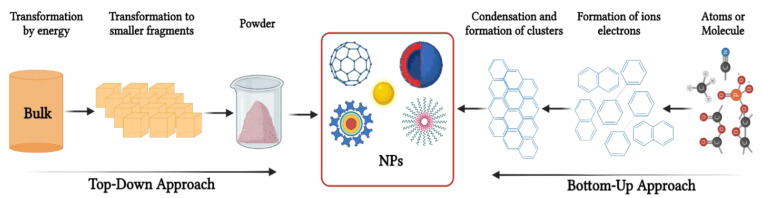
Synthesis of NPs via the bottom-up and top-down approaches. Bulk materials are broken down into small particles using physical methods in the top-down approach, while the bottom-up approach uses wet chemistry to assemble smaller atoms into NPs.

Synthesis of AgNPs, just like other MNPs, can follow the top-down or bottom-up approaches [7,26,27]. The major difference between these two methods is the starting materials involved in the synthesis process. In the top-down approach, the bulk material is used as a starting material, which is then broken down into NPs via various physical and chemical processes, as shown in Figure 1 [4]. In contrast, the bottom-up approach uses atoms as starting material, which is then built up into larger NPs using chemical or green synthesis methods [7].

**Figure 2:**
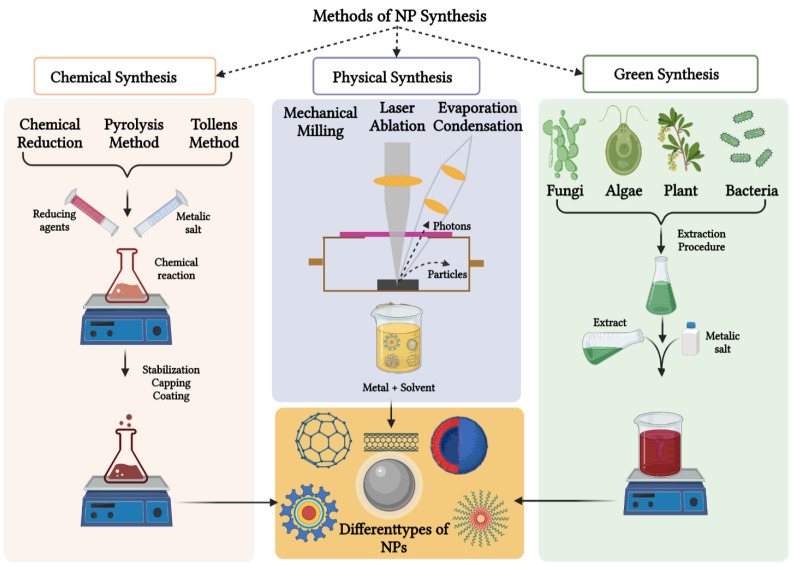
An overview of the synthesis of NPs using the chemical, physical and green synthesis methods. The bulk materials are crushed by physical methods to produce NPs, whereas the chemicals and natural products are used to reduce metal precursors into their respective NPs.

An overview of the physical, chemical, and green synthesis methods used for the synthesis of NPs is highlighted in Figure 2. The physical approach includes methods such as evaporation, condensation, and laser ablation [28]. These techniques are capable of synthesizing larger quantities of AgNPs at high purity in the absence of any chemical-reducing agents [6]. Unfortunately, these physical methods have some drawbacks, such as high power consumption and time-consuming processes [29]. Additionally, complex equipment is required for the synthesis process, thus increasing the operating costs [4].

The chemical synthesis methods use chemicals such as sodium citrate and sodium borohydride to reduce the metal precursors into their respective MNPs. Chemical synthesis methods have been widely used in the production of MNPs by using chemical reducing agents to synthesize their respective MNPs [4]. However, chemical synthesis routes are also associated with drawbacks such as increased toxicity and high reactivity, which pose harmful threats to human and animal health and the environment [30]. Thus, there is a need for an improved synthesis strategy for MNPs in terms of sustainability, eco-friendliness, and non-toxicity. Green synthesis of AgNPs, using bio-reducing agents from natural sources such as microbial and plant extracts, can attain such properties [31].

### 2.1. Green Synthesis of MNPs

The green or biological synthesis methods of MNPs are a cost-effective and eco-friendly alternative to the physical and chemical methods [32]. Another benefit of biological synthesis for MNPs is that waste streams of costly materials such as gold or silver salts can be recycled, ultimately reducing the overall costs of production [30]. Green synthesis makes use of biological or natural entities such as plant or microbial extracts as reducing, capping, and stabilizing agents in the synthesis of NPs.

### 2.2. AgNPs Synthesis Using Microbes

Microorganisms (bacteria, fungi, and yeast) and their components are used in a biological approach to synthesize various MNPs, including AgNPs [14]. The use of microorganisms to synthesize MNPs has been extensively studied for more than three decades, and it has many advantages over chemical and physical methods, including simplicity, low cost, and the use of non-toxic reducing agents. The biomolecules from these microorganisms, such as proteins, polymers, sugars, enzymes, and others, are the ones responsible for reducing and stabilizing the metal precursors to produce biogenic MNPs [33,34].

Microbes can produce AgNPs in two ways through in vitro or in vivo methods. The in vitro method produces NPs through the extracellular process, which usually involves Ag^+^ reduction by cell wall-reducing enzymes or biomolecules secreted in the culture medium. The in vivo method involves the intracellular production of AgNPs with the bio-reduction occurring within the cells [35]. When using microorganisms as a biological source, the growth medium parameters, such as pH, temperature, metal concentration, and exposure time, affect the size and shape of MNPs [30]. Table 1 shows some of the AgNPs synthesized by bacteria and fungi, resulting in different sizes and shapes [36,37]. However, the use of microbes in NP synthesis can be challenging, as both the reducing and stabilizing agents are highly dependent on their growth and maintenance [38]. The AgNPs synthesized using bacteria [36,39] and fungi [37,40] also require rigorous purification steps [41].

### 2.3. AgNPs Synthesis Using Plant Extracts

Plant-mediated synthesis methods provide a sustainable alternative for the synthesis of AgNPs, as plant materials are readily available, eco-friendly, renewable, and affordable [14,42,43]. The phytochemicals in plant (roots, stems, leaves, etc.) extracts are key building blocks in the plant-mediated synthesis of AgNPs [19]. These plant extracts contain an abundance of molecules with carboxyl, amino, carbonyl, hydroxyl, and phenol groups and, thus, have the ability to reduce metals such as silver [44], gold [43], and platinum [45]. Antioxidants are strongly implicated as reducing and capping agents in these processes [46], especially the flavonoids (flavonols, flavan-3-ols), phenolic acids (benzoic, hydroxycinnamic, and ellagic acids) [33], and anthocyanins [16,33].

**Figure 3:**
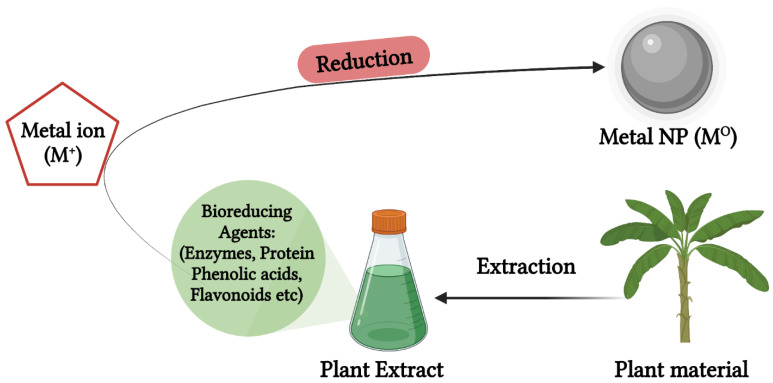
Green synthesis AgNPs using plant extracts. Extracts obtained from various parts of plants are used for bio-reducing and stabilizing AgNPs in a one-step synthesis method.

AgNPs are synthesized using plant extracts in a relatively simple method schematically shown in Figure 3, where the plant extracts obtained from the plant materials (vegetables, fruits, herbs, medicinal plants, etc.) are mixed with an aqueous AgNO_3_ solution in various reaction conditions to produce biogenic AgNPs. The reaction parameters include the type and concentration of the extracts, metal salt concentration, temperature, and pH [47]. In most cases, a color change to brown or yellow signifies the formation of AgNPs. The wide range of phytochemicals present in plant extracts are responsible for the bio-reduction of metal cations, and other plant metabolites such as proteins and chlorophyll are responsible for stabilization of the NPs [47]. The major phytochemicals responsible for the bio-reduction process are aldehydes, ketones, flavones, sugars, terpenoids, carboxylic acids, and amides [27]. Table 2 illustrates bioactive AgNPs synthesized from various extracts obtained from different parts of plant species, such as vegetables [15], fruits [16], and medicinal plants [17,18].

## 3. Biomedical Applications of Biogenic AgNPs

The anti-bacterial efficacy of AgNPs against a diverse spectrum of therapeutically relevant planktonic and sessile pathogenic microorganisms (bacteria, viruses, fungi, and yeasts) has led to tremendous interest in biomedical applications of AgNPs [8] as either therapeutic or drug delivery agents. There have been numerous reports and studies that illustrated that green AgNPs have superior potency and biocompatibility compared with cAgNPs. As a result, the biogenic or green AgNPs have been used in many preclinical and medical applications as anti-microbial, anti-cancer, drug delivery, anti-angiogenesis agents, etc. [8,44]. Although the mode of action of the green AgNPs is not well understood, their anti-bacterial and anti-cancer properties might follow similar mechanisms to that of other MNPs. For example, the induction of reactive oxygen species (ROS) within the cells in both bacteria and cells induces toxicity that ultimately causes their death [8]. Some of these applications are explored herein, and in some cases, compared with cAgNPs to weigh in on their advantages and disadvantages.

### 3.1. Anti-Microbial Applications of Biogenic AgNPs

Antibiotics have been used for many decades to combat infectious diseases. Antibiotics provide a strong baseline for much available modern-day medicine; however, misuse and wrong prescription of antibiotics have lowered their efficiency. These factors have led to the emergence of multi-drug-resistant microorganisms, which have become a worldwide medical concern. As such, the search for non-traditional approaches to combat multi-drug-resistant microorganisms has received increased attention. This has led to the development of novel green nanotechnology-based approaches [49]. AgNPs have been shown to be effective against over 650 microorganisms, including Gram-negative or -positive bacteria, fungi, and viruses. After decades of research, the biogenic AgNPs demonstrated attributes that make them suitable as alternative anti-microbial agents to combat multi-drug resistance [50]. Plant extracts synthesized AgNPs exhibited anti-microbial activities that were comparable and, at times, superior to that of the conventional anti-microbial agents [51]. Additionally, when the green AgNPs were used in combination with the conventional drugs, synergistic effects or improved activity were observed [52].

**Figure 4:**
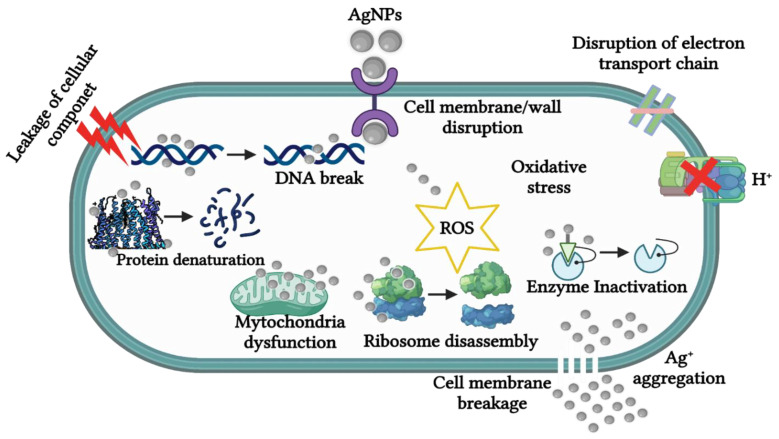
The anti-microbial action of AgNPs. The AgNPs can disrupt and penetrate the bacterial cell wall or membrane. The Ag^+^ ions are then released into the bacterial cell and disrupt the functions of the cellular components and systems, leading to bacterial cell death.

The exact anti-microbial mechanism of the biogenic AgNPs is not fully elucidated [53] and is speculated to follow the four well-defined mechanisms for various nanomaterials, as illustrated in Figure 4. The biogenic AgNPs can adhere to the surface of the cell wall and membrane; penetrate the cell and damage intracellular structures such as the mitochondria, ribosomes, and biomolecules (DNA and protein); induce cellular cytotoxicity and oxidative stress by generating ROS; and lastly, modulate the signal transduction pathway [53]. The AgNPs are perceived to attach to the surface of bacterial cells through electrostatic interaction between the Ag^+^ ions and the negatively charged surface of the cell wall or membrane due to the presence of carboxyl, phosphate, and amino groups. As such, the Ag^+^ ions will then penetrate the membrane, which will, in turn, cause structural and permeability changes leading to the dissipation of the proton motive force and destruction of the cell membrane [51]. Consequently, AgNPs can dissociate and release the Ag^+^ ions into the bacterial cell, which will enhance the anti-microbial activity and cause their death [46].

#### 3.1.1. Anti-Bacterial Activity

At present, many bacterial species from genera, such as *Streptococcus, Pseudomonas, Escherichia, Salmonella,* etc., have developed resistance to many well-known antibiotics, which present major health threats [51]. For example, *Enterococcus faecium*, *S. aureus*, *K. pneumoniae*, *Acinetobacter baumannii*, *P. aeruginosa*, and *Enterobacter* species, collectively termed ESKAPE, are considered the most virulent and classified as high-priority pathogens for human health [49,54]. Medicinal plants have been successfully used in inhibiting the growth of drug-resistant strains, including the ESKAPE pathogens, and thus have the potential to combat anti-microbial drug resistance [55]. Therefore, using these plant extracts in the synthesis of AgNPs provides an attractive strategy to produce alternative anti-bacterial agents that can kill drug-resistant pathogens [50]. The anti-bacterial activity of plant-synthesized AgNPs was somewhat comparable with that of standard antibiotics and, at times, has shown enhanced activity [51]. Moreover, synergistic effects were observed when the plant-synthesized AgNPs were used in combination with the anti-bacterial agents, implying that they can also be used as drug sensitizers [52]. Table 3 summarizes some of the plant-synthesized AgNPs and the type of microorganisms they were tested against [20]. The plant-synthesized AgNPs showed anti-bacterial activity that was comparable to that of the standard antibiotics or drugs such as penicillin-streptomycin, ampicillin, amoxicillin, vancomycin, streptomycin, etc.

#### 3.1.2. Anti-Fungal Activity

Fungal infections have increased at a higher rate and, together with it, the drug-resistant fungi strains, thus requiring more potent anti-fungal agents for treatment. AgNPs have received plenty of attention due to their remarkable anti-bacterial activities, which could also present themselves as promising anti-fungal agents [63]. The anti-fungal activity of green AgNPs is not as extensively studied as that of their anti-bacterial activity. However, the limited studies so far have reported that AgNPs produced from plant extracts possess fungicidal properties. AgNPs from three medicinal plant extracts (*Boswellia ovalifoliolata*, *Shorea tumbuggaia*, and *Svensonia hyderobadensis*) had higher activity against *A. flavus*, *A. niger*, *Curvularia sp.*, *Fusarium sp*., and *Rhizopus sp*. Among these, AgNPs derived from *Svensonia hyderobadensis* showed more activity than AgNPs derived from the other two plants [64]. AgNPs synthesized from stems and flowers of *Teucrium polium* had anti-fungal activity against *Fusarium oxysporum* [65]. *Amaranthus retroflexus*-synthesized AgNPs were reported to have anti-fungal activity against several pathogenic fungal species, especially against *Macrophomina phaseolina* and *F. oxysporum* [66]. AgNPs synthesized from strawberry waste were also reported to have anti-fungal activity against *F. oxysporum*, a plant fungus [67], further demonstrating the importance of AgNPs in both plant and human health.

#### 3.1.3. Anti-Viral Activity

Outbreaks of infectious diseases triggered by newly emerging pathogenic viruses or those that have acquired resistance to currently available anti-viral drugs have prompted the search for novel anti-viral agents [68]. Viral infections are dependent on the virus’s ability to enter and attach to host cells through the binding of viral ligands to the host’s cellular proteins. The best approach for creating new anti-viral agents is to disrupt the interactions between the virus and host cell, thus preventing the virus from attaching and entering the cells. The ideal anti-viral agent should have broad-spectrum activity against pathogenic viral species to be employed as a first-class anti-viral agent against current and future viral epidemics or pandemics. As such, the anti-viral arsenal is in dire need of novel and improved anti-viral agents. AgNPs have emerged as one of the most promising anti-viral candidates, especially since AgNPs have shown broad activity against most microbes [68]. As a result of their unique intrinsic features, AgNPs have shown anti-viral activity against a variety of viruses [69], including HIV-1, monkeypox, hepatitis B, Tacaribe, Rift Valley fever, and influenza (H3N2 and H1N1) [70] viruses. However, the precise anti-viral mechanism of AgNPs, as well as the precise stage of infection at which AgNPs exert anti-viral activity, are still unknown [69].

Preclinical studies have shown that interactions between viruses and NPs result in direct or indirect anti-viral activity. Nanomaterials with indirect activity do not inhibit viruses on their own; instead, they are used as delivery agents to improve the bioavailability of anti-viral treatments and to boost their activity. Furthermore, nanomaterials can elicit an immune response, resulting in either short- or long-term immunity. Nanomaterials with direct action, on the other hand, serve as the active compound and inactivate viruses on their own, most often by modifying the viral structure or genetic material [70].

Three major elements can be derived from previous research on the anti-viral capabilities of AgNPs: (1) AgNPs have shown anti-viral activity against prokaryotic and eukaryotic organisms; thus, making them a viable broad-spectrum anti-viral candidate [71,72]. (2) Smaller AgNPs have higher anti-viral activity in most cases [73,74], and (3) AgNPs generally exert their effect at the early stage of infection of the virus [75].

Biogenic AgNPs were also reported to be potent against several viruses. *Cinnamomum cassia* synthesized AgNPs inhibited the H7N3 virus from infecting the Vero cells [76]. AgNPs synthesized from three medicinal plants, namely *Andrographis paniculata, Phyllanthus niruri,* and *Tinospora cordifolia*, prevented the Chikungunya virus from infecting Vero cells in a dose-dependent manner. The *A. paniculata* AgNPs were the most active, followed by *T. cordifolia* AgNPs. The *P. niruri* AgNPs did not show as significant an inhibitory effect as the other two AgNPs [77], signifying that the phytochemicals indeed influence the function and activity of the AgNPs. AgNPs synthesized from aqueous and hexane extracts of *Lampranthus coccineus* and *Malephora lutea* also prevented infection of Vero cells with HSV-1, HAV-10, and Coxsackie B4 viruses. Furthermore, the *L. coccineus* hexane AgNPs showed higher anti-viral activity against all three viruses, while the *L. coccineus* aqueous AgNPs had weaker anti-viral activity against HSV-1 and no anti-viral activity against HAV-10 and CoxB4 viruses. The *M. lutea* AgNPs showed anti-viral activity against HAV-10 and CoxB4 viruses, with no activity against HSV-1 [78].

### 3.2. Anti-Angiogenesis Activity

Angiogenesis is the process of creating new blood vessels from pre-existing ones [79]. It is required for various physiological processes such as embryo development, ovulation, and wound healing. The combination of several pro-angiogenic and anti-angiogenic factors regulates this process. While the physiological angiogenesis is well controlled, disruptions in this process have been reported in cancer and obesity development and progression, resulting in excessive blood vessel proliferation [80]. The first angiogenesis hypothesis was proposed nearly four decades ago, stating that tumor growth is reliant on its blood vessels to supply nutrients and oxygen to the tumors, for removal of waste and to spread to other tissues [81]; and that cutting off the blood supply can be used as a therapeutic intervention. Strategies targeting factors that contribute to tumor development could successfully treat cancer and other diseases caused by dysfunctional angiogenesis [82].

Vascular Endothelial Growth Factor (VEGF) is one of the well-studied angiogenesis activators which was shown to be overexpressed during tumor growth and metastasis [83]. VEGF expression is upregulated by nuclear factor-kappa B transcription factor, which in turn promotes the expression of anti-apoptotic proteins such as Bcl-2; and prevents cancer cell death [84]. As a result, anti-angiogenesis strategies were used to prevent the development of new blood vessels and supply of blood to the tumor [79] and stop cancer growth and progression [85]. Consequently, anti-angiogenesis strategies inhibited tumor growth, and the tumors were unable to grow larger than 1–2 mm^3^ in size and died as a result of hypoxia [86]. Monoclonal antibodies (mAB) against pro-angiogenic factors or their receptors, matrix metalloproteinase (MMP) inhibitors, and signal transduction blocking are some of the methods that were reported to inhibit angiogenesis. Anti-angiogenic drugs or chemotherapy (Table 4) have been shown to improve cancer outcomes [81]. However, there are several drawbacks associated with the use of angiogenesis inhibitors, including drug resistance, the disruption of VEGF-dependent angiogenesis, and the reduction of radiotherapy response, among others [82]. As such, using monotherapy to prevent angiogenesis could be ineffective [87]. Improvements in the efficacy and biocompatibility of the drugs (angiogenesis inhibitors) were reported when a drug carrier was used. NPs, among others, have done a stellar job in this regard, serving as both a drug carrier and/or angiogenesis inhibitor.

**Figure 5:**
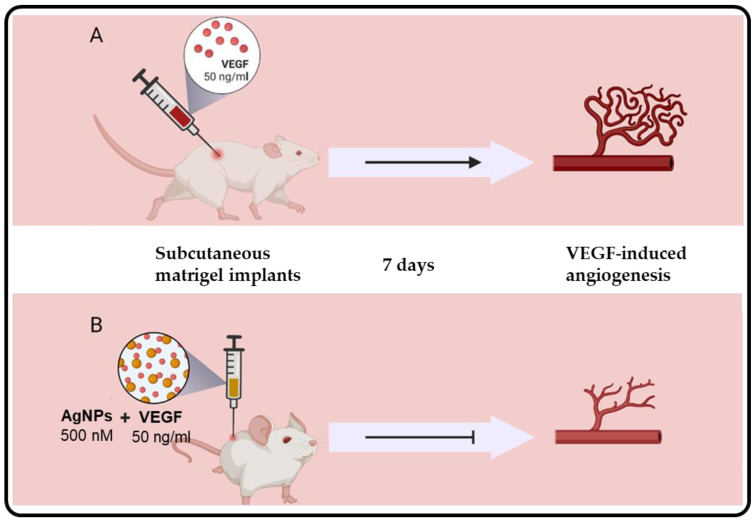
Anti-angiogenic effects of AgNPs in vivo using a matrigel plug model with BRECs. Matrigel plugs containing BRECs with and without 50 ng/mL VEGF were implanted into mice for 7 days. Angiogenesis occurred in mice treated with VEGF alone (**A**). The AgNPs inhibited the VEGF-induced angiogenesis in mice implanted with matrigel plugs containing BRECs with 50 ng/mL VEGF and 500 nM AgNPs after 7 days (**B**). Adapted from [99].

The anti-angiogenic properties of nanomaterials have been reported and provide an alternative candidate for anti-angiogenetic therapy for a variety of diseases [81], including cancers and obesity. NPs may be a useful treatment option as anti-angiogenic or drug-delivery agents. And through antibodies, aptamers, microRNAs, and peptides, among others, the nanomaterials can be targeted to specific tissues [98]. The anti-angiogenic activity of cAgNPs has been reported. In one study, it was associated with the inhibition of hypoxia-inducible factor-1 expression in breast cancer (MCF-7) cells, consequently affecting the expression of anti-angiogenic factors such as VEGF-A and glucose transporter-1 [99,100]. *Bacillus licheniformis* AgNPs inhibited the proliferation and migration of bovine retinal endothelial cells (BRECs) as a model system for angiogenesis after 24 h. The AgNPs activated caspase-3 activity and DNA fragmentation, which in turn inhibited the VEGF-induced PI3K/Akt pathway in BRECs [101]. AgNPs synthesized from *Saliva officinalis* extracts were also shown to have anti-angiogenic activity in vivo. The *Saliva officinalis* AgNPs had a dose dependent anti-angiogenic activity on chick chorioallantoic membranes (CAM). Ross fertilized eggs were exposed to the *Saliva officinalis* AgNPs on day 8. After 4 days, the AgNPs demonstrated dose-dependent anti-angiogenetic effects [80]. Similarly, other plant-extract-synthesized AgNPs reduced blood vessel formation in a CAM assay, these included AgNPs from *Azadirachta indica* leaf [22] and *Ceropegia juncea* [21] extracts. Although the mechanism was not clearly defined, using the matrigel plug model with BRECs in mice, it was shown that AgNPs might induce anti-angiogenic effects by inhibiting the expression of VEGF, as illustrated in Figure 5 [99]. These strategies might also be of health benefit to other diseases with excessive angiogenesis, such as atherosclerosis, arthritis, obesity, pulmonary hypertension, diabetic retinopathy, and age-related macular degeneration [102].

### 3.3. Anti-Cancer Activity

Cancer remains one of the highest killers in the world, with 18.1 million cancer cases reported in 2020. Of these cases, 9.3 million cases were men, and 8.8 million cases were women [103]. The basic problems of early detection and treatment need to be addressed to combat cancer [69]. Moreover, the ability to reach the target site at a sufficient concentration and have efficacious activity without causing harm to healthy cells and tissues are crucial points regarding the effectiveness of anti-cancer drugs [69].

Cancer treatment strategies employ the use of radiation therapy, chemotherapy, surgery, immunotherapy, photodynamic therapy, and stem cell transformation, individually or in combination. However, there are side effects that accompany these treatment strategies, such as non-specificity, limited bioavailability, toxicity, and early drug clearance [104]. The use of chemotherapeutic agents can cause toxic side effects. For example, 5-fluorouracil and doxorubicin, the commonly used chemotherapeutic agents, are linked to renal toxicity, cardiotoxicity, myelotoxicity, and blood vessel constriction [104].

Research into newer technologies to prevent systemic and bystander side effects, as well as to improve on the existing drugs, has led to the development of nanotechnology-based therapeutics [105]. Doxil (doxorubicin encapsulated in liposomes) [106] and Abraxane (paclitaxel bound with albumin) [107] are the first organic nano-formulations to be FDA-approved for the treatment of cancer. The AuNPs were the first MNPs to be approved for human trials as drug delivery and diagnostic agents [1,108]; AgNPs followed suit as anti-microbial agents [109]. Several studies have demonstrated the potential anti-cancer activity of AgNPs, including AgNPs synthesized from various plant extracts [23]. Table 5 highlights some of the plant-synthesized AgNPs and their anti-cancer effects on multiple cancer cell lines, namely breast (MCF-7) [110], lung (A549) [111], cervical (HeLa) [112], colon (HT-29, Caco-2) [113], prostate (PC-3) [26], and VCaP [114] cancer cell lines. The half-maximal inhibitory concentration (IC_50_) of AgNPs was comparable to that of standard anti-cancer drugs [23] and a validation that AgNPs could be useful as anti-cancer agents.

The working principles and mechanisms of action of AgNPs are critical and unique to each cell type. Many factors influence their cytotoxicity and genotoxicity, including size, shape, surface charge, surface coating, solubility, concentration, media, surface composition, distribution into cells, method of entry, and cell type [122]. Their biological activities are also dependent on their possible interaction with proteins in cell culture media or in vivo, forming a protein corona that might enable interactions between AgNPs and cells to induce or ameliorate toxicity [123]. AgNPs can penetrate the cell through the assistance of cellular components, such as the endosomes and lysosomes, or through passive or active transport modes, such as pinocytosis, phagocytosis, etc. [124].

The interaction of AgNPs with biomolecules can cause a variety of physiological and metabolic changes, including oxidative stress, conformational changes, enhanced membrane permeability, mutations, signaling pathway activation, ionic exchange disorder, and exposure to novel protein epitopes [125]. Several in vitro models demonstrated that ROS-mediated toxicity is more apparent and results in cellular and metabolic changes. As such, oxidative stress is the most likely mechanism of AgNPs-induced toxicity [126]. The formation of peroxide (H_2_O_2_) and superoxide (O_2_) radicals changes the trans-membrane potential of mitochondria, allowing the respiration mechanism to become uncoupled, thus causing oxidative stress [127]. ROS is one of the mechanisms commonly followed by plant-based AgNPs; it alters the signal transduction pathway and induces cell death by apoptosis [128,129,130]. AgNPs trigger intrinsic apoptosis by releasing cytochrome c into the cytosol and translocation of Bax to the mitochondria, as well as cell cycle arrest in the G1 (cell growth) and S (DNA synthesis) phases [131]. Plant-based AgNPs are also capable of inducing cell death by upregulating the expression of genes that causes apoptosis, such as the p53, Bax, and poly-ADP ribose polymerase (PARP)-1, p21. *Mentha arvensis* AgNPs activated breast cancer cell death following different pathways, indicating the ability of green AgNPs to overcome drug resistance. *Mentha arvensis* AgNPs induced caspase-3-dependent death in MDA-MB-231 cells due to mutations in p53, and through caspase 9-dependent apoptosis in p53-expressing MCF-7 cells [132].

### 3.4. Anti-Diabetic Activity

Diabetes is a chronic disease in which blood sugar levels rise due to inadequate insulin output or when the cells do not respond well to insulin. As a result, diabetes can be either insulin-dependent or insulin-independent [52]. Several enzymes are involved in this complex disease, with two of the most important regulators being α-glucosidase, which is responsible for the breakdown of disaccharides to monosaccharides, and α-amylase, which is responsible for the breakdown of the long-chain carbohydrates (polysaccharides to disaccharides). Thus, inhibition of these enzymes would provide an effective anti-diabetic strategy [51].

The anti-diabetic effect of plant-synthesized AgNPs has been reported for both in vitro and in vivo studies. In vitro, the potential of the AgNPs to reduce blood glucose levels was tested based on their ability to block the secretion of the two enzymes [52] and, in some instances, the dipeptidyl peptidase IV [133]. Some of the plant-based AgNPs able to inhibit the activity of the three enzymes are summarized in Table 6. AgNPs synthesized from *Calophyllum tomentosum* [133], *Punica granatum* [134], *Ficus palmata* [24], and *Lonicera japonica* [135] leaf extracts demonstrated α-amylase inhibition (AAI) and α-glucosidase inhibition (AGI) in vitro. The green AgNPs were found to have high levels of AAI and AGI; as such, the green synthesized AgNPs might have strong anti-diabetic activity [24,25,134,136]. In vivo, the anti-diabetic activity of these AgNPs was measured based on their ability to reduce blood glucose levels and their effect on other blood biochemical events (cholesterol and triglycerides). The *Solanum nigrum* AgNPs reduced blood glucose levels in alloxan-induced diabetic rats [136]. *Eysenhardtia polystachya* AgNPs also protected the pancreatic β cells from oxidative cellular damage, enhanced secretion of insulin and hypolipidemia. These effects were also replicated in a glucose-induced diabetic zebrafish [137]. *Cassia auriculata* AgNPs, in a similar manner, were shown to reduce blood glucose levels and normalized other markers associated with diabetes in streptozotocin-induced diabetic rats [138].

### 3.5. Anti-Inflammatory Activity

Inflammation is a vital defensive mechanism in which the body reacts to external stimuli such as bacteria, irritants, or damaged cells. Various cells, including molecular mediators, immune, and blood cells, are involved in this complicated process [139]. Chronic inflammation, on the other hand, can also do more damage than good and can last for longer periods. Prolonged inflammation is a major problem for many metabolic diseases such as obesity, insulin resistance, and diabetes [140,141], as well as in wound healing. It is classified as the second phase of the wound-healing process [10,142]. As such, the quest for compounds that can stop chronic inflammation may be beneficial in the treatment of these diseases and in human health.

AgNPs are among the MNPs that were demonstrated to have anti-inflammatory properties [10]. AgNPs have shown anti-inflammatory response during wound healing by modulating the expressions of various inflammatory response genes in vitro such as tumor necrosis factor (TNF)-α, interferons, interleukin 1, cyclooxygenase (COX)-2, and matrix metalloproteinases-3 [27,133,143,144]. A number of plant-based AgNPs with anti-inflammatory effects that are beneficial to wounds have been reviewed elsewhere [142] and show potential for the treatment of metabolic and autoimmune disorders. *C. orbiculata* AgNPs inhibited pro-inflammatory cytokines (TNF-α, IL-6, and IL-1β) in THP-1 differentiated macrophages and NK cells [17]. *Piper nigrum* AgNPs selectively inhibited the expression of IL-1β and IL-6 [145], suggesting the anti-inflammatory activity of these green AgNPs. Another anti-inflammatory activity was demonstrated by the *Viburnum opulus* L fruit AgNPs on skin keratinocytes (HaCaT). The IL-1α was released due to irradiation of the cells indicating the anti-inflammatory activity of the AgNPs. The phenomenon was further investigated in vivo in carrageenan-induced hind paw edema and found that injecting AgNPs into the skin of Wistar rats moderated cytokine release within 2 h. The AgNPs demonstrated inhibitory activity against COX-2, which is one of the main causes of inflammation [146]. Overall, these studies demonstrated that green-synthesized AgNPs could be used as a valuable therapeutic method for the treatment of inflammation.

### 3.6. AgNPs as Drug Delivery Agents

Drug delivery via MNPs is an effective strategy for the treatment of a variety of diseases [147]. The two most important strategies for effective drug delivery systems are: slow and sustained drug release, and delivery to specific targets. These conditions can be met through active or passive delivery methods [148]. Conventional strategies for cancer treatment, such as chemotherapy and radiotherapy, come with various side effects, such as drug toxicity, lack of specificity, unpredictability, and drug resistance [149].

Since one of the most studied topics for improving current human healthcare practice has been the precise and selective delivery and action of therapeutic agents, NPs have received a lot of attention when it comes to the design and development of novel and improved drug-delivery systems [8]. More specifically, green synthesized AgNPs could overcome the disadvantages associated with conventional therapies by reducing their side effects and enhancing their efficacy [149]. Green nanotechnology is an innovative approach that can improve disease treatment, and when green AgNPs are coupled with anti-cancer drugs can directly deliver the drugs to the tumor tissues due to the ability of the AgNPs to pass through various biological barriers [149]. The size of the NPs influences drug uptake and distribution in the cells via endocytosis. AgNPs synthesized from the *Aerva javanica* extract coupled with the anti-cancer agent (gefitinib) had higher apoptotic potency than gefitinib alone on the MCF-7 cells. Gefitinib delivery by the AgNPs improved the drug efficacy while reducing the negative effects [150].

Outside of oncology, AgNPs conjugated with the anti-seizure drugs against brain-eating amoebae (*Naegleria flowleri*) were used to treat central nervous system infection. Anti-seizure drugs (diazepam, phenobarbitone, and phenytoin) that are known to pass through the blood-brain barrier were added on the surface of AgNPs as capping agents and demonstrated general anti-amoebic activity against both trophozoite and cyst phases. The AgNPs-drug conjugates showed a considerable increase in fungicidal activity against both trophozoite and cyst amoebic stages, as compared with the drugs alone [4].

## 4. Perspectives and Concerns for Clinical Application of AgNPs

Nanotechnology is gaining worldwide recognition due to the growing capacity and applications of engineered NPs [151]. Over the last few decades, the wide distribution and use of nanomaterials in medical imaging, disease diagnosis, drug delivery, and a variety of consumer-based products have been on the rise. This is attributed to their novel properties [44,152], which are influenced by their small size, large surface area to volume ratio, drug loading capacity, and ease of functionalization of the NP surface. However, the extensive application of these nanomaterials has raised concerns regarding potential health and environmental effects [151]. Despite the numerous advantages offered by the NPs, the potential health hazards cannot be overlooked due to their uncontainable release into the natural environment and possibly deleterious effects on organisms that are continually subjected to exposure to these nanomaterials [153]. Hence, toxicity studies are perpetually conducted to make the application of NPs more environmentally friendly. Among the most frequently studied nanomaterials are fullerenes, carbon nanotubes, gold, titanium oxide, iron oxide, and silver NPs [151].

The increase in the number of commercial AgNPs-based products without any regulatory restraints and risk assessment policies is a major issue that can turn into a health scare. The lack of proper health hazard assessments is mainly due to the historical use of silver-based products for health purposes, which must be regarded as a call for concern because prolonged use of silver-based compounds in wound care was associated with argyria, bacteria resistance, and toxicity. Since these were topically administered, the effects were easy and quick to observe [10]. However, unlike the bulk silver compounds, AgNPs are smaller in size, and their route of administration can differ based on the intended application. All these might alter their uptake, distribution and ultimately their biological effects. Studies have reported on the size, shape, surface composition, and dose of the AgNPs as the major contributing factors to their toxicity; some external factors include time, suspension media, cell, and species type [11,154].

AgNPs have demonstrated a remarkable potential for the development of novel anti-microbial, biosensing, imaging, drug-delivery, and therapeutic agents [8] and are used to improve or as alternatives to the current therapies [155]. The incorporation of AgNPs within the biomedical sector [10,109,156] is attributed to their biocompatibility and anti-bacterial activity. Several cAgNPs-based formulations were approved for clinical trials by the FDA (Table 7) as anti-microbial and wound healing agents [10] for topical and oral applications in humans [109]. In fact, 37 AgNPs-based clinical trials are registered in the U.S. National Library of Medicine ClinicalTrials.gov database to date. The majority of these products were for dental and wound healing applications, although there were some registered for rhinosinusitis, pain management and COVID-19. The completion of 51% of the trials validates that AgNPs have been ingested, inhaled, or absorbed by human beings, including children from 1–12 years of age. Their incorporation in dental composite resins, such as restorative, prosthetic, endodontics, implantology, and periodontology, has undoubtedly shown notable anti-microbial properties and reduced microleakage in the root canal system. When used in dental irrigation, AgNPs-based formulations showed similar potency to that of sodium hypochlorite against *E*. *faecalis* [157]. Nonetheless, exposure to AgNPs can be lethal even at low levels [154], and these detrimental effects can be long-lasting and passed to future generations [11]. In animals, administration of AgNPs exposed them to vital organs such as the kidneys, lungs, spleen, and liver, and their accumulation in these organs has been associated with an altered expression of genes involved in oxidative stress, apoptosis, and ion transport [158]. Although the biogenic AgNPs are considered to have a reduced risk of toxicity compared with cAgNPs, they are also non-selective. The toxicity of biogenic AgNPs is also influenced by a number of parameters, among others being the size [113].

Colloidal stability is considered a significant factor affecting the fate of AgNPs. When introduced into the environment, AgNPs possess a high affinity for biomolecules, such as DNA, proteins; leading to the formation of AgNPs-protein corona, which can alter the activity of the NPs. Furthermore, AgNPs can be affected by pH, where if the environmental pH corresponds to the isoelectric point (pI) of biomolecules on the AgNPs, the NPs become unstable and ultimately form aggregates [159]. The release of Ag^+^ ions into the environment greatly impacts the toxicity of AgNPs [12]. Importantly, their fate after being released into the environment and interaction with the surroundings is of serious concern [13]. The most commonly known process related to AgNPs transformation is oxidative dissolution [12] when subjected to oxygen-rich conditions [13]. The capping agents in the green AgNPs, which act as stabilizers, might protect the AgNPs against agglomeration and reduce their cytotoxicity. The biomolecules on the surface of the AgNPs are likely to enhance their biocompatibility and render them safe for use in various biomedical applications [160].

## 5. Conclusions

Plant-extract-synthesized AgNPs can be used as an alternative to cAgNPs and conventional treatment strategies, as well as in combination with current treatments to enhance their efficacy. Due to their size-dependent physicochemical properties, eco-friendliness, availability, and cost-effectiveness; green AgNPs have been used in a variety of biomedical applications, such as anti-bacterial, anti-fungal, anti-viral, anti-cancer, anti-angiogenesis, anti-inflammatory, and drug delivery agents. There is a large amount of research describing the benefits of green AgNPs in novel biomedical strategies. As such, their long- and short-term toxicity requires a much more thorough investigation to alleviate the fear of their societal and environmental impacts. This review demonstrated the versatility of green AgNPs in many innovative therapeutic strategies that can help fight against current and future pandemics due to their multifaceted activities.

## Figures and Tables

**Table 1:** AgNPs synthesized from various bacteria and fungi species.

Microbes	Strain	AgNPs Size (nm)	AgNPs Shape	References
Bacteria	*Arthrospira indica*	48−67	Spherical	[39]
	*Pseudomonas mandelii*	1.9−10	Spherical, irregular	[36]
Fungi	*Penicillium expansum*	14−25	Spherical, irregular	[37]
	*Aspergillus niger*	25−175	Spherical	[40]

**Table 2:** AgNPs synthesized from various plant extracts and algae cultures.

Species	Type	Plant Source	Hydrodynamic Size (nm)	References
Plants	*Allium cepa*	Onion	5–80	[15]
	*Solanum lycopersicum* L.	Tomato	2–50	
	*Acacia catechu*	*Acacia catechu* powder	5–80	
	*Cotyledon orbiculata*	Plant leaves	100–140	[17]
	*Pyrus communis* L. cultivars	Fruit pulp and skins	110–190	[16]
	*Terminalia mantaly*	Root, stem bark, leaves	11–83	[18]
Algae	*Coelastrum* sp.	Algae cultures	19.2	[48]
	*Spirulina* sp.		13.85	
	*Botryococcus braunii*		15.67	

**Table 3:** Plant-synthesized AgNPs and their anti-bacterial activity.

Plant Material	Plant Extract	Test Bacteria	Shape of AgNPs	Size of AgNPs (nm)	References	
*Curcuma Longa*	Turmeric powder extract	*E. coli* *Listeria monocytogenes*	Mostly spherical with quasi-spherical, decahedral, ellipsoidal, and triangular shapes	5–35	[56]	
*Pyrus communis* L. cultivars	Fruit peel and pulp	*S. aureus, MRSA*, *P. aeruginosa, E. coli*	Spherical	110–190	[16]	
*Terminalia Mantaly*	Stem bark, leaves, and roots	*S. aureus, Streptococcus pneumoniae, K. pneumoniae, Salmonella enterica, Shigella flexneri, Hoemophilus influenza*	Polydispersed	11–83	[18]	
*Salvia Africana Lutea*	Leaves	*Staphylococcus epidermidis, P. aeruginosa*	Polygon and spherical	25–40	[57]	
*Sutherlandia frutescens*	Leaves	*S. epidermidis, P. aeruginosa*	Spherical	200–400	[57]	
*Sapindus mukorossi*	fruit pericarp extract	*S. Aureus*, *P. aeruginosa*	Spherical	≤30	[58]	
Grape fruit	peel extract	*E. coli*, *S. aureus*, *Enterococcus faecalis*	-	0–100	[59]	
*Areca catechu*	fruits extract	*E*. *faecalis, Vancomycin-resistant E*. *faecalis, P. aeruginosa*, Multidrug-resistant *P. aeruginosa, Acinetobacter baumannii,* Multidrug-resistant *Acinetobacter baumannii*	Spherical	100–300	[20]	
*Ipomoea aquatica*	leaf extract	*Salmonella, Staphylococcus* sp., *E. coli*	Spherical	5–30	[60]	
*Acacia* lignin	wood dust	*Bacillus subtilis, Bacillus circulans*, *S. aureus*, *E. coli, Ralstonia eutropha, P. aeruginosa*	Spherical	2–26	[61]	
*Amaranthus Tricolor L.*	Red spinach leaf extract	*E. coli*	Spherical	5–40	[62]	

**Table 4:** Anti-angiogenic agents and some of their inhibitory strategies.

Anti-Angiogenesis Drugs	Angiogenesis Inhibitory Strategies	References
Bevacizumab (Avastin)	Target VEGF and inhibits formations of VEGF complexes such as VEGF-A and VEGF-2	[88]
Semaxanib, Sunitinib, Sorafenib, Vatalanib	Inhibition of receptor tyrosine kinase	[89,90,91]
GEM 220	Inhibition of VEGF	[92,93]
Endostatin	Inhibition of endothelial-cell survival	[94]
Erlotinib, Gefitinib	Inhibitors of EGFR	[95]
Celecoxib, Rofecoxib	COX-2 inhibitors	[96,97]

**Table 5:** Plant extract-synthesized AgNPs with anti-cancer activity.

Cancer	Plant	AgNPs Size (nm)	AgNPs Shape	Cell Line	IC_50_ (μg/mL)	References
Breast cancer	*Achillea biebersteinii*	12	Spherical, pentagonal	MCF-7	20	[80]
	*Melia dubia*	7.3	Irregular		31.2	[110]
	*Ulva lactuca*	56	Spherical		37	[115]
Liver cancer	*Cucumis prophetarum*	30–50	Polymorphic shape (ellipsoidal, irregular)	HepG-2	94.2	[116]
Lung cancer	*Rosa damascene*	15–27	Spherical	A549	80	[117]
	*Gossypium hirsutum*	13–40	Spherical		40	[118]
	*Syzygium aromaticum*	5–20	Spherical		70	[111]
Cervical cancer	*Podophyllum hexandrum*	14	Spherical	HeLa	20	[112]
	*Heliotropium indicum*	80–120	Spherical	Siha	20	[119]
	*Azadirachta indica*	2–18	Triangular and hexagonal		≤4.25	[120]
Colon cancer	Gum arabic	1–30	Spherical	HT-29 Caco-2	1.55 1.26	[113]
Prostate cancer	*Alternanthera sessilis*	50–300	Spherical	PC-3	6.85	[26]
	*Gracilaria edulis*	55–99	Spherical	PC-3	53.99	[121]
	*Dimocarpus longan*	8–22	Spherical	VCaP	87.33	[114]

**Table 6:** Plant-synthesized AgNPs with anti-diabetic activity.

Plant Type	Core Size (nm)	Hydrodynamic Size (nm)	AgNPs Inhibitory Effect	Test Sample	References
*Calophyllum tomentosum* leaves extract	-	24	α-amylase	Starch	[133]
			Dipeptidyl peptidase IV	Gly-Pro-P-Nitroanilide	
			α-glucosidase	4-nitrophenyl-α-d glycopyranoside	
*Punica granatum* leaves	20–45		α-amylase	Starch	[134]
		35–60	α-glucosidase	para-nitrophenyl-α-D-glucopyranoside	
Grape Pomace	5–40	-	inhibits α-amylase and α-glucosidase	α-amylase and α-glucosidase	[25]
*Solanum nigrum*	4–25	-	Glucose inhibition	alloxan-induced diabetic rats	[136]
Bark of *Eysenhardtia polystachya*	5–25	36.2	Promote pancreatic β-cell survival; Restores insulin secretion in INS-1 cells	Glucose-induced adult Zebrafish (hyperglycemia)	[137]

**Note**: -, not reported.

**Table 7:** AgNPs-based products approved by FDA for clinical trials.

Status	Study Title	ClinicalTrials.gov Identifier	AgNPs Formulation	Condition	Participants	Administration Route	Phase
Active, recruiting	Colloidal silver, treatment for COVID-19	NCT04978025	AgNPs	Severe acute respiratory syndrome	50	Oral and inhalation	N/A
Completed	Topical application of silver nanoparticles and oral pathogens in ill patients	NCT02761525	12 ppm of AgNPs- innocuous gel	Critical illness	50	Oral mucosa	N/A
	Silver nanoparticles in multi-drug-resistant bacteria	NCT04431440	AgNPs	Methicillin and vancomycin-resistant *S*. *aureus*	150	Tested on clinical isolates	N/A
	Nano-silver fluoride to prevent dental biofilms growth	NCT01950546	5% nanosilver fluoride (390 mg/mL 9 nm AgNPs, 21 mg/mL chitosan, 22 mg/mL NaF)	Dental caries	30	Applied on cervical vestibular surfaces of incisors and canines	1
	The anti-bacterial effect of nano-silver fluoride on primary teeth	NCT05221749	Nano-silver fluoride	Dental caries in children (1–12 years old)	50	Oral	3
	Evaluation of silver nanoparticles for the prevention of COVID-19	NCT04894409	ARGOVIT^®^ AgNPs (Mouthwash and nose rinse)	COVID-2019	231	Mouthwash and nose rinse	N/A
	Assessment of postoperative pain after using various intracanal medications in patients with necrotic pulp	NCT03692286	AgNPs and calcium hydroxide vs. AgNPs in gel form	Necrotic pulp (postoperative pain)	30	Intracanal medication	4
	Effect of thyme and carvacrol nanoparticles on aspergillus fumigatus isolate from intensive care patients	NCT04431804	AgNPs	Aspergillosis	210	Aspergillus isolates	N/A
	Evaluation of diabetic foot wound healing using hydrogel and nano silver-based dressing vs. traditional dressing	NCT04834245	Hydrogel and nano silver-based dressing	Diabetic wounds	30	Topical wound dressing	N/A
	Comparison of central venous catheters (CVC) with silver nanoparticles versus conventional catheters	NCT00337714	CVC impregnated with AgNPs (AgTive^®^)	CVC related infections	472	Cannulation	4
Unknown	Topical silver nanoparticles for microbial activity	NCT03752424	AgNPs-cream	Fungal infection (Tinea pedis, Capitis and Versicolor)	30	Topical	1
	Research on the key technology of burn wound treatment	NCT03279549	Nano-silver ion gel and dressings	Burns	200	Topical	N/A
	Addition of silver nanoparticles to an orthodontic primer in preventing enamel demineralisation adjacent brackets	NCT02400957	AgNPs incorporated into the primer orthodontic Transbond XT	Tooth demineralisation	40	Dental application	3
	Fluor varnish with silver nanoparticles for dental remineralisation in patients with Trisomy 21	NCT01975545	25% of 50 nm AgNPs in fluor varnish	Dental remineralisation in patients with Down syndrome	20	Oral varnish	2
	Efficacy of silver nanoparticle gel versus a common anti-bacterial hand gel	NCT00659204	Nano-silver gel (SilvaSorb^®^ gel)	Healthy	40	Topical on hands	3

## Data Availability

Not applicable.
